# Utilization of postnatal care for newborns and its association with neonatal mortality in India: An analytical appraisal

**DOI:** 10.1186/1471-2393-12-33

**Published:** 2012-05-09

**Authors:** Abhishek Singh, Awadhesh Yadav, Ashish Singh

**Affiliations:** 1 Department of Public Health & Mortality Studies, International Institute for Population Sciences, Mumbai, 400 088, India; 2 International Institute for Population Sciences, Mumbai, 400 088, India; 3 Indira Gandhi Institute of Development Research, Mumbai, 400 065, India

**Keywords:** Postnatal care for newborns, Kangaroo care, Neonatal mortality, Matched case–control Study, Conditional logit regression, India

## Abstract

**Background:**

39% of neonatal deaths in India occur on the first day of life, and 57% during the first three days of births. However, the association between postnatal care (PNC) for newborns and neonatal mortality has not hitherto been examined. The paper aims to examine the association of PNC for newborns with neonatal mortality in India.

**Methods:**

Data from District Level Household Survey, waive three (DLHS-3) conducted in 2007–08 is utilized in the study. We used conditional logit regression models to examine the association of PNC with neonatal mortality. The matching variables included birth order and the age of the mother at the birth of the newborn.

**Results:**

The findings suggest no association between check-up of newborns within 24 hours of birth and neonatal mortality. However, the place where the newborns were examined was significantly associated with neonatal mortality. Moreover, findings do reveal that children of mothers who were advised on ‘keeping baby warm (kangaroo care) after birth’ during their antenatal sessions were significantly less likely to die during the neonatal period compared to those children whose mothers were not advised about the same.

**Conclusions:**

The findings are relevant because ‘keeping baby warm’ is one of the most cost-effective and easiest interventions to save babies from dying during the neonatal period. Though randomized controlled trials have already demonstrated the effectiveness of ‘keeping baby warm’, for the first time this has been found effective in a large-scale population-based study. The findings are of immense value for a country like India where the neonatal mortality rates are unacceptably high.

## Background

Although there is no doubt that India has improved in terms of infant and child survival; from a infant mortality rate of 80 per 1000 live births in 1990 to 55 in 2007 [1,2] and under-five mortality rate of 109 per 1000 live births in 1991–92 to 74 in 2005–06 [3]; one cannot deny the fact that despite this progress, current infant mortality rates in India are alarmingly high compared to other countries of similar socio-economic conditions. Data from a recently conducted household survey in India suggests a neonatal mortality rate of 39 per 1000 live births and a postneonatal mortality rate of 18 per 1000 live births [3] – with significant state level and urban–rural differentials. Trends in infant mortality rates indicate that the declines in infant mortality have stagnated in the recent past in most of the states in the country [4] – rather at unacceptably high levels. This is despite the fact that Government of India has launched a number of interventions such as Child Survival and Safe Motherhood (CSSM) Programme, Reproductive and Child Health Programme (RCH), National Rural Health Mission (NRHM), etc. to arrest persisting high levels of neonatal and postneonatal mortality.

The highest risk of death for both newborns and mothers occurs around the time of childbirth and the immediate postpartum period becomes extremely critical both for mother and baby [5]. More than two-thirds of newborn deaths occur by the end of the first week after birth, with up to one-half of all deaths occurring in the first 24 hours of birth [6]. India is no exception to this: 39% of neonatal deaths in India occur on first day of life, and 57% during the first three days [7].

Promoting antenatal care (ANC) and skilled attendance at birth is clearly not enough for improving child health. Strategies that promote universal access to PNC have been recommended for some years [8] and have the potential to contribute to sustained reductions in neonatal and maternal mortality [5]. Small-scale studies clearly highlight the contribution of postnatal care strategies, including ‘*kangaroo care*’ in reducing neonatal mortality in low income settings [9-14]. Although the provision of PNC has been an important component of various governmental interventions in India [15], however – given the focus on skilled attendance at birth and antenatal care – the PNC component has received little attention.

Studies documenting the utilization of PNC in India are extremely limited. We could come across only two studies that document PNC in the Indian context [16,17]. However, none of the above studies examined association of PNC with neonatal mortality. Though it is important to examine the association between PNC use and neonatal mortality, it has not hitherto been investigated in a population-based representative study. The present study, therefore, investigated the role of PNC in improving neonatal survival using a nationally representative, large-scale population-based dataset in India. Given a new survey data collected in 2007–08 which collected more information on PNC we were able to examine the research problem in bit detail.

## Methods

### Data

We use data from the ‘District Level Household Survey’ round three (DLHS-3) conducted in 2007–08 in 601 districts spread over 34 states and union territories of India. DLHS-3 was based on a multi-stage stratified systematic sampling design which resulted in national and state-representative samples after applying weighting factors to control for complex survey design [18]. The main instrument for collection of data in DLHS-3 was a set of structured questionnaires. In all 643,944 ever married women aged 15–49 years and 166,260 unmarried women aged 15–24 years were interviewed in the survey. Information on postnatal care was sought from all women who had given birth in the five years preceding the survey date irrespective of whether they delivered at home or at a health facility. This is unlike the previous DHS surveys where the information on postnatal care was sought only from women who delivered in a health facility. The availability of this additional information in DLHS-3 provided us an opportunity to investigate the association between PNC and mortality during the neonatal period.

One of the important survey instruments in DLHS-3 was the women questionnaire which collected detailed information on birth history (births since January 1, 2004), health, breastfeeding, place of delivery, mode of delivery, and related information for mothers and children. The birth history data provided an opportunity to examine the association between PNC and neonatal mortality in India.

### Outcome and exposure variables

The outcome variable of interest in the analysis is mortality during the neonatal period. Neonatal mortality is defined as the probability of dying in the first month of life. The exposure variables of interest in the analysis are measures of PNC – whether the newborn received any check-up within 24 hours of birth and whether the newborn was examined in a government/private health facility. These explanatory variables were computed in order to investigate the association between PNC and mortality during neonatal period. Since, no direct question on ‘whether attempts were made to keep baby warm’ was asked in the DLHS-3, we utilized information on ‘whether mother was advised on keeping baby warm during one of her antenatal sessions’ as a proxy of whether baby was kept warm or not. This proxy measure of PNC is used as the third explanatory variable in the analysis.

### Control variables

Besides PNC, other socio-demographic variables have also been shown to have a significant impact on mortality during the neonatal period – so accordingly we also included woman’s education, wealth status, place of delivery, maternal complication during delivery (yes, no), mode of delivery (normal, c-section), initiation of breastfeeding (initiated breastfeeding within an hour, initiated breastfeeding later), place of residence (urban–rural), geographic region of residence (north, central, east, northeast, west and south), and sex of the newborn in the analysis.

### Statistical analysis

The analysis is guided by the framework for analyzing a matched case–control study [19,20]. The newborns that died before completing the first month of life were treated as *cases* and those who survived the first month after birth were treated as *controls*. We examined the utilization of PNC for newborns among the cases and the controls. If PNC was significantly less in *cases* than in *controls*, then we concluded that PNC was associated with neonatal mortality. Several studies in the past have used such framework to examine association between risk factors and mortality during infancy [21-24]. Conditional binary logistic regression models (that are appropriate for analyzing matched case–control studies) were also estimated to assess the adjusted effect of PNC on the likelihood of neonatal mortality [25-28]. 3588 neonatal deaths were reported in DLHS-3. We utilized ‘k_1i_:k_2i_’ matching with k1i > 1 for at least one group, which is available in STATA 11.0 software. We utilized the *clogit* command in STATA 11.0 which fits models appropriate for all matching schemes or for any mix of the schemes because the matching k_1i_:k_2i_ can vary from group to group [29]. This was actually done to utilize the full sample of births available in DLHS-3 dataset. The matching variables utilized in the conditional logistic regression models were ‘birth order of the newborn’ and the ‘age of the mother at the time of birth of the newborn’. Regression results were adjusted for aforementioned socioeconomic and demographic characteristics.

We excluded the births that took place in one month preceeding the survey date to account for the censoring in the analysis. We also excluded 1672 babies (0.79% of the study sample) that died within the first few hours and minutes of birth from our analysis because the detailed questions on postnatal care were not applicable to those births. The question asked on postnatal care in DLHS-3 was: ‘Did your child have any check-up after delivery within 24 hours of birth?’ The response categories to this question were *yes*, *no*, *child did not survive*. The subsequent questions on postnatal care were not asked to women who reported *child did not survive* as the answer to the above question.

We estimated three separate sets of conditional logit models and included one of the PNC variables at a time as the independent variable along with the other control variables. The study samples utilized in the three models were 201762, 196585, 145662 births respectively. All the independent variables were tested for possible multi-collinearity before putting them into the regression models.

## Results and discussion

Results from the survey indicate that only 48% of the newborns received any PNC check-up within 24 hours of birth (Table 1). Around 64% of the babies did, however eventually receive two or more check-ups within the first 10 days after birth. As expected, a majority of these babies were examined in a private facility (56%). Moreover, only 50% of the mothers received advice on ‘keeping baby warm’ during one of their antenatal sessions.

**Table 1:** Utilization of postnatal care services for babies born in the five years preceding the survey, 2007-08

**Service**	**Percentage**	**Number**
*During ANC, mother received any advice on keeping baby warm*		
Yes	49.6	76,717
No	50.4	77,952
*Newborn received any PNC check-up within 24 hours of birth*		
Yes	48.3	101,323
No	51.8	108,655
*Number of PNC check-ups in first 10 days after birth*		
Less than two check-ups	36.3	34,480
Two or more check-ups	63.7	60,563
*Place of first PNC check-up*		
Government facility	44.3	42,122
Private facility	55.7	53,008

Results presented in Table 2 clearly suggest that a higher percentage of mothers of surviving neonates were advised on *keeping baby warm* during their antenatal sessions compared to the mothers of neonates who died (50% versus 37%). Newborns that died during the neonatal period were less likely to have received check-up within 24 hours of birth compared to newborns that survived the neonatal period (45% versus 48%).

**Table 2:** Percentage of mothers receiving advice on keeping baby warm during ANC session, percentage of newborns receiving any check-up within 24 hours of birth and percentage of newborns receiving check-up in government/private facility by the survival status of children, 2007-08

**Service received**	**Newborn experienced mortality during neonatal period**	
	**Yes**	**No**
During ANC, mother received advice on keeping baby warm	37.0 (871)	49.8 (75,846)
Newborn received any check-up within 24 hours of birth	44.6 (945)	48.3 (100,379)
Newborn was checked in a government facility	44.6 (323)	44.3 (41,798)
Newborn was checked in a private facility	55.4 (402)	55.7 (52,606)

Note: The numbers in the parentheses are the absolute numbers.

The conditional logistic regression results are presented in Table 3. Findings do not suggest any association between ‘check-up within 24 hours of birth’ and mortality during neonatal period indicating that there was no significant difference in the chances of dying during the neonatal period between the newborns who received any check-up within 24 hours of birth and those who didn’t. However, the place of check-up was associated with mortality of the newborns during the neonatal period. Newborns that were examined in a government facility were indeed less likely to die during the neonatal period than the newborns that were not examined at all (the odds ratio was 0.79; 95% CI: 0.67-0.92). The relationship between the place of check-up and the neonatal mortality was also significant in the ‘Wald Test’, thus suggesting that the place of check-up was indeed associated with better chances of survival during the neonatal period for the newborns.

**Table 3:** Results of conditional logistic regression models assessing the role of PNC in mortality during neonatal period, 2007-08

Covariate/category	Mortality during neonatal period
*Newborn received any check-up within 24 hours of birth*^*a*^	
Yes ^®^	
No	0.96 (0.86,1.07)
*Newborn was checked in*^*‡, b*^	
No check-up ^®^	
Government facility	0.79* (0.67,0.92)
Private facility	0.90 (0.79,1.03)
*During ANC, mother received advice on keeping baby*^*c*^*warm*	
Yes ^®^	
No	1.27* (1.13,1.43)

® reference category.

* *p* < 0.05, values in the parentheses are 95% confidence intervals.

‡ Wald Test was significant at *p* < 0.05 in case of mortality during neonatal period.

a 291 groups (or 5120 observations) were dropped because of all positive or all negative outcomes.

b 288 groups (or 6424 observations) were dropped because of all positive or all negative outcomes.

c 281 groups (or 7231 observations) were dropped because of all positive or all negative outcomes.

Matching variables: Birth order of the newborn and the age of mother at the birth of the index newborn.

Control variables: Mother’s education, wealth status, place of residence, region of residence, place of delivery of the index newborn, maternal complication at the time of delivery, mode of delivery, initiation of breastfeeding, and sex of the newborn.

Interestingly, newborns whose mothers were not advised on *keeping baby warm* during any of their antenatal sessions were 1.27 (95% CI: 1.13-1.43) times more likely to die during the neonatal period compared to the newborns whose mothers were advised to do so. This finding clearly indicates that advising mothers on *keeping baby warm* is likely to be beneficial for neonatal survival in India.

## Conclusions

Our paper, for the first time, has investigated the association between PNC and neonatal mortality in India using a nationally representative population-based dataset. The analysis was carried out using the framework of a matched case–control study. Surprisingly, the ‘check-up of newborns within 24 hours of birth’ was not associated with neonatal mortality. However, the choice of facility where the newborn was examined was indeed associated with neonatal mortality – newborns examined in a government facility were significantly less likely to have died during the neonatal period than those who were not examined. But newborns examined in a private facility were no less likely to have died during the neonatal period than those who were not examined at all. Findings have important policy lessons to be learnt. Just promoting skilled birth attendance is clearly not enough to address high levels of neonatal mortality in India as a majority of newborns die in the first few weeks of life. Strategies must focus on making PNC for newborns accessible to all. Findings clearly suggest that utilization of PNC for newborns is extremely limited and has not picked up in the past two decades. Government of India in the recent past has launched conditional cash transfer scheme more commonly known as ‘*Janani Suraksha Yojana (JSY)*’ to promote institutional deliveries in India. Recent evaluations of the scheme suggest that the scheme has been quite effective in increasing antenatal care and facility births in the country [30,31]. Now the time has come when Government of India must also think of such schemes for promoting PNC for newborns and mothers as well.

Interestingly, this paper, using a nationally representative population-based dataset in India, also provides the first empirical evidence on the association between simple and cost-effective interventions like *keeping baby warm* or *kangaroo care* and neonatal mortality. Findings indeed suggest that newborns whose mothers were not advised on *keeping baby warm* were significantly more likely to die during neonatal period compared to those newborns whose mothers were advised about the same. Our findings lend support to the findings of various small-scale studies [9-14] that have demonstrated the important role of ‘*kangaroo care*’ in saving lives of newborns. Though the *advice on keeping baby warm* was only a proxy for actual behaviour, the findings are indicative. Advising pregnant women on *keeping baby warm* during antenatal or postnatal sessions is likely to save lives of a number of newborns during the crucial first few days of birth. If advised properly, women are more likely to adopt any intervention that can save the lives of their newborns, provided the intervention is within their reach and is acceptable under their socio-cultural setting. ‘*Kangaroo care*’ is one such intervention which is not likely to face any opposition, either from the communities in general or social and religious institutions in particular.

The strengths and limitations of our study should also be noted. The main advantage of this study concerns usage of rich, reliable, and large-scale population-based dataset (DLHS-3). The large sample size allowed us to examine the associations between PNC and neonatal mortality in detail. DLHS surveys are conducted under the stewardship of Ministry of Health and Family Welfare (MoHFW), Government of India, with the objective to monitor the performance of the Reproductive and Child Health Programme in India [18]. More than 99% of India’s population is represented in DLHS-3 [18]. Survey teams receive formal training and quality control measures are in place. The data from the DLHS is of optimal quality and studies in the past have found this data comparable with other large-scale surveys conducted in India [32]. Another strength of the study is the systematic use of matched case–control design to establish association between PNC and neonatal mortality. Though our study has a number of advantages, it also has a few limitations. Although, we adjusted our results for a number of socio-economic, demographic and residence related variables in the conditional logit models, we could not control for birth weight, gestational age, and birth interval owing to the non-availability of these in the DLHS-3 dataset. Studies have shown that these are well-known risk factors for neonatal mortality. Furthermore, we had to exclude 1672 births from the analysis due to non-availability of information on PNC. The exclusion of these births should not distort the associations established in the study because the newborns need to survive for a few hours for PNC to take place. Also these births accounted for only 0.79% of the study sample. While we note these limitations, the advantage of using a matched case–control approach in our view significantly outweighs the potential limitations associated with the available data. Furthermore, the findings of our study provide additional and more conclusive evidence on the importance of postnatal care for newborns for reducing neonatal mortality in a low-resource developing country like India.

If India has to achieve millennium development goals 4 (MDG 4), promotion of such cost-effective interventions is the need of the hour. The Government of India has taken a number of initiatives under the ambitious National Rural Health Mission (2005–2012) for improving the health of the rural masses. Under this Mission, the Government is investing a lot of efforts and resources for improving the rural public health infrastructure and to make public health system accessible to all, irrespective of their socio-economic status. Simple and cost-effective intervention like ‘*kangaroo care’* is unfortunately often omitted in the national policies and programme documents. Special focus on interventions like ‘*kangaroo care*’ along with the architectural corrections in public healthcare system is likely to pay immediate and higher dividends than just carrying out the architectural corrections in the public healthcare system. Promoting such interventions is simple (and cost free), given the recruitment of an Accredited Social Health Activist (ASHA) in almost every village of India under the National Rural Health Mission. ASHA along with her colleagues associated with other Government sponsored programmes, can be entrusted with the promotion of such interventions in the community in general and among pregnant women in particular.

## Competing interests

The authors declare that they have no competing interests.

## Authors’ contributions

AS conceptualized the paper and finalized the analysis plan; AY analyzed the dataset and generated the tables; AS and AshishS prepared the first draft of the paper. All authors read and approved the final manuscript.

## Pre-publication history

The pre-publication history for this paper can be accessed here:

http://www.biomedcentral.com/1471-2393/12/33/prepub
